# Progress of the National Insurance Bill

**Published:** 1911-08-05

**Authors:** 


### HOSPITALS AND STATE INSURANCE.

### PROGRESS OF THE NATIONAL INSURANCE BILL.

### THE POSITION OF THE VOLUNTARY HOSPITALS DISCUSSED.

The position of hospitals under the proposed National Insurance scheme was fully discussed when the Committee stage of the Bill was resumed on Monday. The discussion arose on Clause 12 which contains the provisions in the case of insured persons who are inmates of hospitals, etc., one of which is that no payment shall be made on account of sickness or disablement benefit to an insured person during such period and that the benefit shall be applied in whole or in part for the relief or maintenance of his dependents, if any.

#### Objection to Clause 12.

Mr. Pollock moved an amendment with the object of eliciting further information from the Chancellor, and in the debate which followed the leading part was taken by Mr. Austen Chamberlain. Mr. Chamberlain urged that the hospitals could not expect to receive the same amount of support from the public in future. Where the hospitals are in the main supported by endowments they will have much less to fear, but "in those places where the Hospital Saturday collections have been largest they may expect to lose more, for when you have compulsorily levied a charge of fourpence a week upon every workmen it is hardly to be expected that he will voluntarily devote an additional one penny per week, as he has been accustomed to do in the works of many of our large towns, to the support of institutions of the character which we are now discussing." Mr. Chamberlain went on to say that it was calculated that forty-four hospitals in the Midland Counties showed that between 50 and 60 per cent. of their entire income was liable to be adversely affected by the Bill, and in many individual cases of hospitals, within that area, the proportion so affected rose up to 75, 80, and even 90 per cent. His view was that when an insured person was treated in a voluntary hospital the whole of his sick pay, if he had no dependent, should go towards his maintenance in the hospital, and if he had any dependents the money which was due to him should be divided between the hospital and the dependents. As an alternative he suggested that the Chancellor might take a portion of the money which is now allocated for sanatoria and allow it to be allotted to hospitals by some impartial central authority.

#### The Chancellor of the Exchequer Replies.

Mr. Lloyd George expressed the opinion that the alarm generally and naturally felt by those who are engaged in hospital work had no foundation in fact. The first thing that would happen under the Bill would be that hundreds of thousands of out-patients would be transferred from the charge of the hospitals to the charge of the insurance fund. In the second place, the money provided for the treatment of tuberculosis would relieve hospitals to a considerable extent. As to the possible diminution of subscriptions, he did not see why the workmen should subscribe less in future. Nor did he think employers would subscribe less, one of his reasons for holding this view being that the Workmen's Compensation Act, which threw heavy charges on employers, had not had the effect of diminishing subscriptions to hospitals. As to new sources of revenue, he thought that if there were a danger of hospitals being closed, or any number of beds being closed, it would be to the interests of Friendly Societies to subscribe.

#### Further Discussion.

Mr. Lawson, Treasurer of the Hospital Saturday Fund, was less optimistic than the Chancellor; there might be a decrease in the number of out-patients, but he pointed out that the out-patients cost comparatively little — namely,

2s. 2d. compared with £5 10s. He was convinced that the work in connection with in-patients would be enormously increased, and his opinion was that it should be obligatory on the part of Health Committees to pay for the work done in the hospitals. The debate was continued by Mr. Jonathan Samuel, Mr. Cassel (who pointed out that the effect of the clause was to deprive the insured person of the right to make a contract with the hospital in respect of the expenditure actually incurred in his maintenance), Captain Clive, Mr. Lansbury (who said they could not give public money to hospitals unless there was some sort of public control over the expenditure of the money), Mr. Hume Williams, Mr. O'Grady, Mr. Lyttelton (who agreed with Mr. Cassel that the best plan was to leave it to the hospital to agree with the insured person or his society as to what proportion of the sick pay should go to the hospital), Mr. Harwood, Sir R. Finlay, Mr. Hamar Greenwood, Sir Henry Craik, Mr. C. Bathurst, Mr. Baird, Mr. Peto, Mr. Ellis Griffith, the Attorney-General, and Mr. Jowett, at the conclusion of which Mr. Pollock's amendment was withdrawn.

### The Division of Sick Pay.

Mr. Austen Chamberlain then moved an amendment providing that the sick pay due to an insured person should be divided between the hospital and his dependents, if any, in such proportions as the local Health Committee might determine. Dr. Addison hoped the Committee would not agree to take money for the sake of the hospitals from the dependents of persons in hospitals. He agreed that the hospitals must be paid for services rendered to insured persons, and he thought that an organisation working through the local Health Committee was the proper way to deal with the hospital part of the scheme. The discussion was continued by other speakers and the amendment was withdrawn.

### Tuesday's Discussion.

When consideration of the clause was resumed on Tuesday Mr. Lloyd George moved an amendment providing that if an insured person is an inmate of a hospital or infirmary supported by charity and has no dependents his sick pay shall, "if an agreement for the purpose has been made with the hospital or infirmary," be paid in whole or in part according to such agreement towards the maintenance of such person in the hospital or infirmary. Provided that in the case of a married woman or widow who is entitled to sickness benefit in addition to maternity benefit no part of the maternity benefit shall be paid or applied for the relief or maintenance of her dependents, but may be paid to the hospital or infirmary of which she is an inmate as aforesaid in like manner as if she had no dependents." This was a modification of an amendment which had been standing in the Chancellor's name for some days. During the discussion of this amendment it was agreed, on the motion of Mr. Austen Chamberlain, to insert the words "or convalescent home" after the word hospital, and after further discussion the Chancellor's amendment with that addition was carried. It is interesting to note that in the course of his speech Mr. Lloyd George said it seemed to him very undesirable that hospitals should be dependent upon the precarious kind of income on which they had now to rely, but he did not think it was desirable to attempt to deal generally with the position of hospitals in this Bill. Mr. Lyttelton agreed that it would be dangerous to take away the voluntary character of hospitals, but the effect of this Bill would be to increase the obligations of hospitals and to diminish their revenues.

### Dr. Addison's Amendment Passed.

On Clause 13, which provides that the benefits shall be administered by approved societies or local Health Committees, an amendment moved by Dr. Addison providing for the transference of the administration from the societies to the local Health Committees was carried by an overwhelming majority, the Chancellor having recommended the Committee to agree to the amendment. As this amendment comprises one of the most important principles embodied in the claims of medical practitioners, there was much satisfaction among those members who have been representing the case for practitioners at the result.

